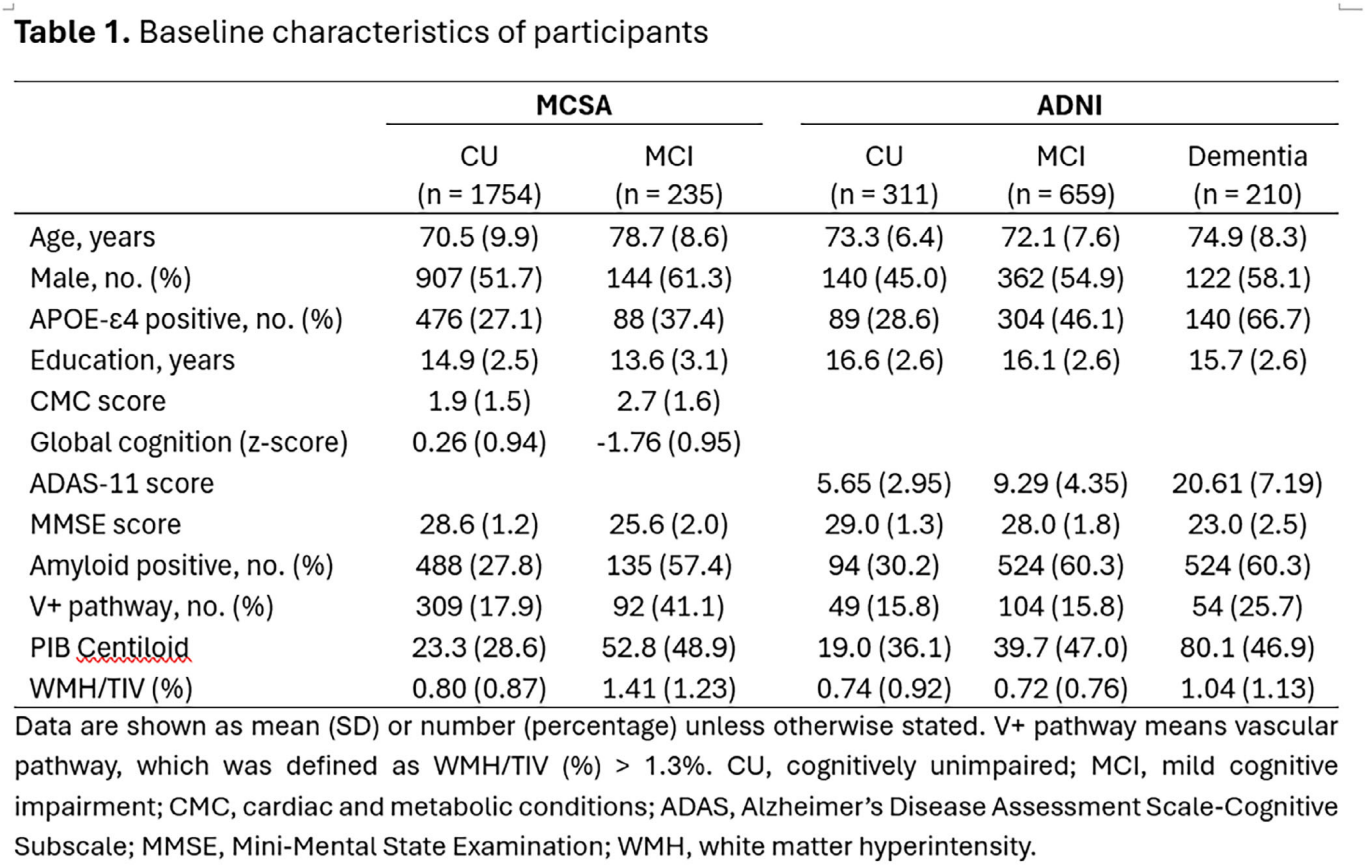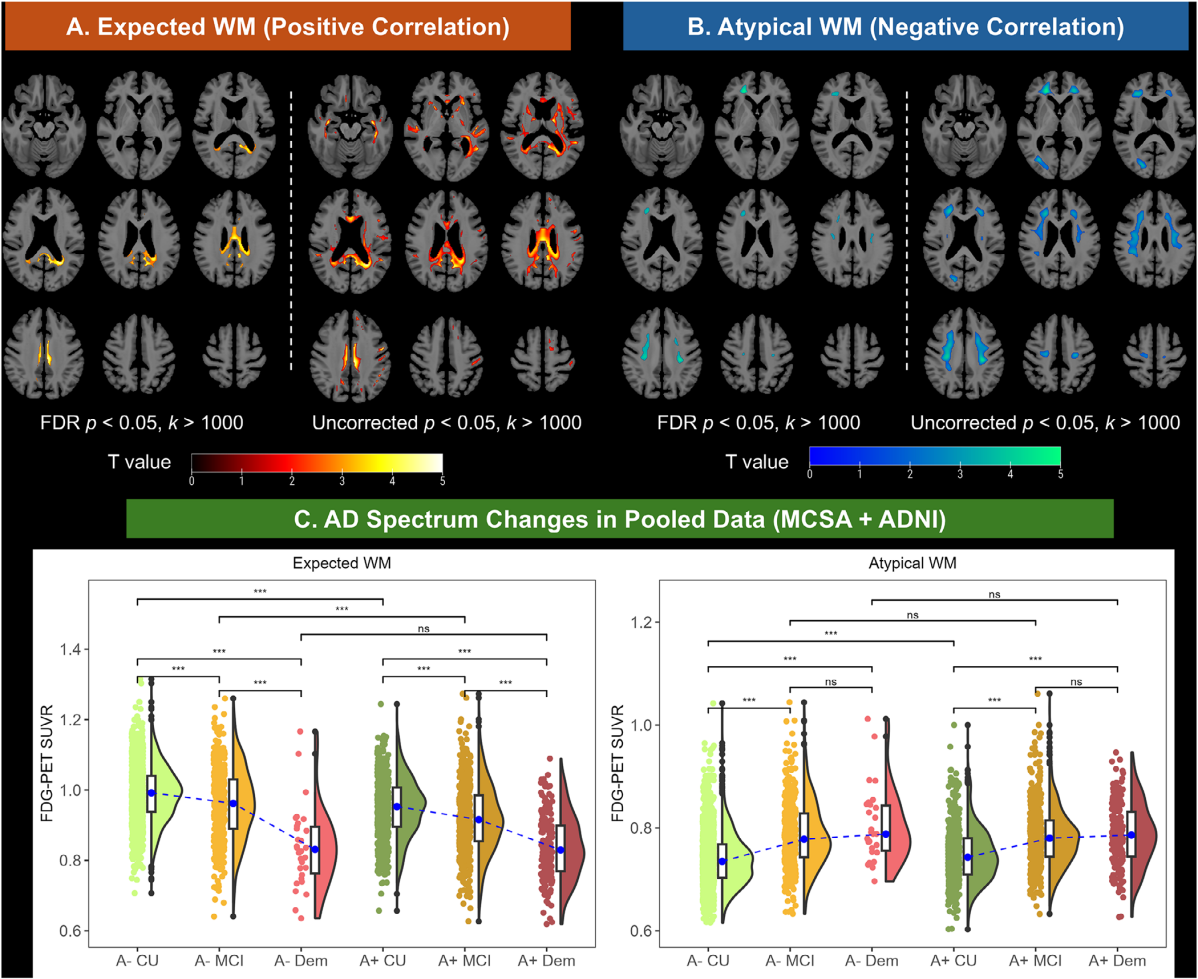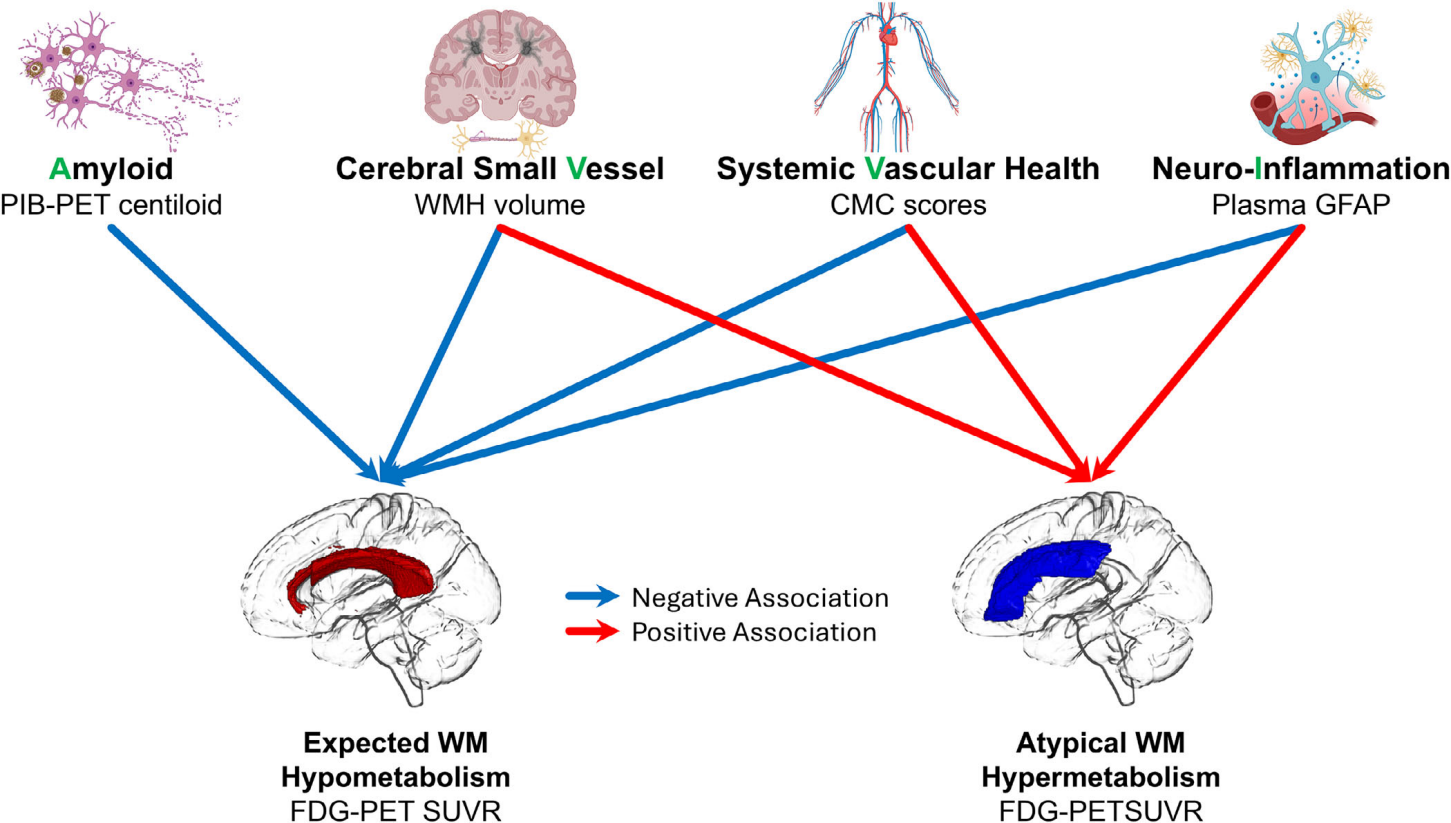# Increased Metabolism in Corona Radiata as a Signature of Lower Cognitive Resilience in Aging and Alzheimer's Disease

**DOI:** 10.1002/alz70856_103799

**Published:** 2025-12-24

**Authors:** Wen Zhang, Sheelakumari Raghavan, Jianqiao Tian, Scott A. Przybelski, Heather J. Wiste, Matthew L. Senjem, Christopher G Schwarz, Robert I. Reid, Mary M. Machulda, Ronald Petersen, Jonathan Graff‐Radford, Clifford R. Jack, Val J Lowe, Prashanthi Vemuri

**Affiliations:** ^1^ Department of Radiology, Mayo Clinic, Rochester, MN, USA; ^2^ Department of Radiology, Nanjing Drum Tower Hospital, Affiliated Hospital of Medical School, Nanjing University, Nanjing, Jiangsu Province, China; ^3^ Mayo Graduate School of Biomedical Sciences, Rochester, MN, USA; ^4^ Department of Quantitative Health Sciences, Mayo Clinic, Rochester, MN, USA; ^5^ Department of Health Sciences Research, Mayo Clinic, Rochester, MN, USA; ^6^ Mayo Clinic, Rochester, MN, USA; ^7^ Department of Psychiatry and Psychology, Mayo Clinic, Rochester, MN, USA; ^8^ Department of Neurology, Mayo Clinic, Rochester, MN, USA

## Abstract

**Background:**

Aging and dementia are generally associated with lower metabolism in gray matter on FDG‐PET and have been widely studied. However, white matter (WM) metabolic changes remain understudied. We investigated the metabolic changes in WM associated with aging and dementia and their relationships with established Alzheimer's Disease (AD) biomarkers and cognitive outcomes.

**Method:**

We analyzed data from 3,169 participants (72 ± 9 years; 53% male; 65% cognitively unimpaired) enrolled in the Mayo Clinic Study of Aging (MCSA) and the Alzheimer's Disease Neuroimaging Initiative (ADNI). All participants underwent baseline MRI, amyloid‐PET, and FDG‐PET scans, as well as neuropsychological evaluations, with at least one clinical follow‐up. Voxel‐wise multiple regression analysis identified WM metabolic changes associated with global cognition in cognitively unimpaired MCSA participants (*n* = 1,754). Univariate analyses and structural equation models were applied to elucidate the driving factors underlying the WM metabolic changes in MCSA data. Multiple regression and linear mixed‐effects models evaluated the explanatory utility of WM metabolism for baseline global cognition and cognitive decline in both cohorts. In a subset of MCSA participants, we evaluated neurite density index from diffusion MRI as a surrogate of WM health and glial fibrillary acidic protein (GFAP) as a surrogate of neuroinflammation to understand underlying mechanisms.

**Result:**

We identified an atypical WM signature (AWM‐Signature) in the anterior and superior corona radiata, where glucose metabolism increased as cognitive performance worsened (Figure 1). Hypermetabolism in AWM‐Signature was related to increased age, amyloidosis, vascular disease, and neuroinflammation, as well as decreasing WM neurite density in the same regions (Figure 2). Hypermetabolism in AWM‐Signature regions predicted faster future cognitive decline, even after accounting for demographics, amyloid, and vascular disease metrics. The results observed in the AWM‐Signature regions were consistently in the opposite direction of the associations seen with gray matter and typical WM regions.

**Conclusion:**

We found unexpected metabolism increases in corona radiata, a hub that carries significant portion of the brain's neural traffic, with aging and disease which likely indicates a compensatory response. These changes may be useful cognitive resilience measures and require further investigation to understand their role in maintaining cognitive function.